# Variability in screening prevention activities in primary care in Spain: a multilevel analysis

**DOI:** 10.1186/s12889-015-1767-5

**Published:** 2015-05-07

**Authors:** Magdalena Rosell-Murphy, Teresa Rodriguez-Blanco, Julio Morán, Mariona Pons-Vigués, Josep M Elorza-Ricart, Jordi Rodríguez, Clara Pareja, María Ángeles Nuin, Bonaventura Bolíbar

**Affiliations:** Institut Universitari d’Investigació en Atenció Primària Jordi Gol (IDIAP Jordi Gol), Av Gran Via de les Corts Catalanes, 587, 08007 Barcelona, Spain; Equip d’Atenció Primària Serraparera. Institut Català de la Salut, Cerdanyola del Vallès, Spain; Universitat Autònoma de Barcelona, Bellaterra (Cerdanyola del Vallès), Spain; Dirección Atención Primaria, Servicio Navarro de Salud – Osasunbidea, Navarra, Spain; SIDIAP, Institut Universitari d’Investigació en Atenció Primària Jordi Gol (IDIAP Jordi Gol), Barcelona, Spain; Equip d’Atenció Primària La Mina. Institut Català de la Salut, Barcelona, Spain

**Keywords:** Primary health services, Primary health care, Primary prevention, Electronic health records, Multilevel analysis, Clinical practice variations

## Abstract

**Background:**

Despite evidence of the benefits of prevention activities, studies have reported only partial integration and great variability of screening in daily clinical practice. The study objectives were: 1) To describe Primary Health Care (PHC) screening for arterial hypertension, dyslipidaemia, obesity, tobacco use, and excessive alcohol consumption in 2008 in 2 regions of Spain, based on electronic health records, and 2) To assess and quantify variability in screening, and identify factors (of patient, general practitioners and PHC team) associated with being screened, that are common throughout the PHC population.

**Methods:**

Multicentre, cross-sectional study of individuals aged ≥16 years (N = 468,940) who visited the 426 general practitioners (GPs) in 44 PHC teams in Catalonia and Navarre in 2008. Outcomes: screening for hypertension, dyslipidaemia, obesity, tobacco use, and excessive alcohol consumption. Other variables were considered at the individual (sociodemographics, visits, health problems), GP and PHC team (region among others). Individual and contextual factors associated with the odds of being screened and the variance attributable to each level were identified using the SAS PROC GLIMMIX macro.

**Results:**

The most prevalent screenings were for dyslipidaemia (64.4%) and hypertension (50.8%); the least prevalent was tobacco use (36.6%). Overall, the odds of being screened were higher for women, older patients, those with more comorbidities, more cardiovascular risk factors, and more frequent office visits, and those assigned to a female GP, a GP with a lower patient load, or a PHC team with a lower percentage of patients older than 65 years. On average, individuals in Navarre were less likely to be screened than those in Catalonia. Hypertension and dyslipidaemia screenings had the least unexplained variability between PHC teams and GPs, respectively, after adjusting for individual and contextual factors.

**Conclusions:**

Of the studied screenings, those for obesity, tobacco, and alcohol use were the least prevalent. Attention to screening, especially for tobacco and alcohol, can be greatly improved in the PHC setting.

**Electronic supplementary material:**

The online version of this article (doi:10.1186/s12889-015-1767-5) contains supplementary material, which is available to authorized users.

## Background

Many chronic diseases that contribute to mortality burden, such as cardiac disease, diabetes and cancer, are mostly preventable [1]. National and international organizations have long published recommendations and clinical guidelines for prevention activities in Primary Health Care (PHC), based primarily on vaccinations, early detection of diseases, screening activities, and control of risk factors [2-8].

In 1988, the Spanish Society of Family and Community Medicine (semFYC) launched the Preventive Activities and Health Promotion Program (PAPPS) to promote the implementation of preventive and health promotion services in PHC. Its recommendations have been adopted throughout Spain [9,10]. Furthermore, the PHC characteristics of accessible, longitudinal, and comprehensive care [11] provide an ideal setting for prevention activities with broad population impact [8].

Nonetheless, despite evidence of the related benefits, national [10,12-14] and international [15-19] studies have reported only partial integration of prevention activities in daily clinical practice, and observed great variability in their implementation. These studies have identified patient, health professional, and PHC team factors that contribute to this variability.

The creation of PHC databases from health records makes available an abundance of rich information [20]. Although some of these computerized databases show underreporting of prevention activities [21,22], they are currently the most thorough, essential information source about health care activity. These databases allow studies of large representative populations, homogeneous data collection, information-gathering at the individual level, longitudinal studies, and assessment of the variability in clinical practice [23]. However, in Spain as well as in other countries, studies of the implementation of preventive services and their variability were hampered by a lack of homogeneity between the databases.

In 2006, the Registry of Preventive Services in Primary Care (REGIPREV) was created from electronic health records to collect the available data on prevention activities by PHC teams from different regions of Spain, and to analyse their implementation [24]. The objective of this study was to describe PHC screening for arterial hypertension, dyslipidaemia, obesity, tobacco use, and excessive alcohol consumption in 2008, in the 2 regions, Navarra and Catalonia, that were the first REGIPREV participants. It also aimed to assess and quantify screening variability and to identify related factors common to the general PHC population that could account for differences between general practitioners (GPs) and PHC teams. In-depth knowledge of these factors will help to develop strategies to increase prevention activities and reduce variability, thereby improving the planning and administration of PHC services [25,26].

## Methods

This multicentre, cross-sectional study involved 44 PHC teams in Catalonia and Navarre (about 7.4 and 0.6 million inhabitants, respectively). In Catalonia, teams were selected from those pertaining to the Catalan Institute of Health (covering 80% of the Catalan population). Inclusion criteria at the PHC team level were computerization of medical records by January 1, 2005 and majority agreement (>80%) among a team’s professionals to participate in the study. Random cluster sampling was stratified by region, with the PHC team as the unit of randomization. At the GP level, we excluded those with non-normal (>3000 or <400) patient lists. Individuals aged ≥16 years assigned to participating GPs and who visited their PHC centre at least once in 2008 were included. Exclusion criteria were individuals with more than 350 visits per year and, for analysis of obesity screening, patients younger than 18 years (to avoid pubertal growth in estimating obesity prevalence). The study protocol and sample size calculation has been previously published [26].

Data were drawn from the REGIPREV database of encrypted and anonymized information on the individuals assigned to the participating GPs. An algorithm was applied to extract equivalent data from the health records software used in each region (“Atenea” in Navarre and “ECAP” in Catalonia). The research team matched the ICD-10 codes used in Catalonia to Navarre’s ICPC-2 codes and constructed a table of equivalencies based on that established by WONCA [27,28]. Information on GP and PHC team characteristics was obtained from paper or online questionnaires completed by each GP and by team directors.

### Variables

Dichotomous dependent variables were set to positive if they met the following PAPPS criteria [29]:

- *Arterial hypertension screening*: 1 recorded systolic and diastolic blood pressure measurement within the past 2 years.
- *Dyslipidemia screening*: 1 recorded measurement of total cholesterol in men aged 16 to 35 years, women aged 16–45 years, and patients older than 75 years; for all other patients, 1 recorded measurement within the past 5 years.
- *Obesity screening*: 1 recorded body mass index (BMI) value within the past 4 years.
- *Tobacco screening*: smoking status recorded within the past 2 years.
- *Alcohol screening*: alcohol consumption recorded within the past 2 years.

In each case, screening was analyzed for the population without the risk factor of interest.

Independent variables were considered at 3 levels:

- *Individual*: age (<45, ≥45 - <65, ≥65 years), sex (male/female), number of health problems (0–3, 4–6, 7–11, ≥12) and number of PHC visits in 2008 (1–2, 3–5, 6–10, ≥11). The number of health problems was used as a morbidity indicator; it was calculated as the sum of the number of different health problems (coded by ICPC-2) recorded up to 31 December 2008. Number of visits and number of health problems were categorized as quartiles of the visited study population. The following risk factors were considered: hypertension (yes/no), dyslipidaemia (yes/no), obesity (BMI ≥30 = yes), smoking (smoker, exsmoker, nonsmoker), and at-risk drinker (standard beverage unit >28/week for male and >17/week for female = yes).
- *GP*: age (years, continuous, centred at its grand mean), sex (male/female), teaching mentor (yes/no), and the following continuous variables: years with the same patient list, total number of assigned individuals, coverage (percentage of assigned patients visited during the study period), and percentage of assigned patients older than 65 years. The last three variables were obtained by aggregating data by GP.
- *PHC team*: region (Navarre/Catalonia), type (urban when the PHC centre is located in a city with ≥15,000 inhabitants; rural when it covers 2 or more villages smaller than 10,000 inhabitants; and semiurban otherwise), teaching centre (yes/no), years of electronic health records available (years, continuous) and adherence to PAPPS program (yes/no). Adherence to the PAPPS program is voluntary, with a minimum set of preventive activities required to qualify; a periodic evaluation is carried out [9,10]. Economic incentives to GPs was not considered as an independent variable at the team level because all PHC teams in each region had the same incentives (in Navarre, incentives were related to all studied screenings; in Catalonia, only to alcohol). Therefore, this factor was taken into account in the “region” variable.

### Statistical analysis

Descriptive statistics were used to summarize overall information. The SAS PROC GLIMMIX macro in SAS 9.3 [30,31] was used to identify individual and contextual factors associated with the odds of being screened and to estimate the variance attributable to each level. This macro fit multilevel logistic regression models, assuming a binomial distribution and a logit link function. We included random effects at the GP and PHC team levels to account for possible correlations within clusters. To explore contextual phenomena that might differ in magnitude for different groups of people [32], random slopes were used to determine whether a PHC team or GP context modified the association between individual characteristics and screening. We used a variance components covariance structure.

We modeled individuals (level-1 units) as nested within 426 GPs (level-2 units) and GPs nested within 44 PHC teams (level-3 units). First, models were fitted with no covariates at any level (i.e., only the intercept, empty model), to assess whether there was significant variation at each level. All models showed significant random effects for all levels. Hierarchical models were developed by sequentially adding the above-mentioned groups of variables to the empty models, as follows: 1) we estimated the effect of individual-level characteristics in the outcomes and allowed the effect of these covariates to vary by GP and team (i.e., allowing for level-2 and level-3 random slopes); 2) we added the GP-level covariates, plus the average number of health problems per GP (as a continuous covariate centred at its PHC team mean); 3) finally, we added the team-level covariates and also included the average number of patients, average number of health problems, average coverage, and average percentage of patients older than 65 years for all GPs in each PHC team (as continuous covariates centred at their grand mean). Only the significant covariates were included in the final models. We checked for significant variances with the Wald test [33,34]. The proportional change in variance (PCV) was calculated.

Analyses were performed using SAS statistical software, v.9.3 (SAS Institute Inc, Cary, NC) and IBM SPSS statistics (PASW Statistics), v.20.0 (SPSS Inc, Chicago, Ill).

The study was approved by the IDIAP Jordi Gol Ethics Committee. Since confidentiality was ensured by data encryption and anonymization on REGIPREV database, written informed consent for participation in the study was not necessary.

## Results

In the 44 selected PHC teams, 426 GPs had a total of 468,940 assigned individuals, 61.5% of them in Catalonia and 38.5% in Navarre. Patients’ mean age was 49 years (47.1% younger than 45 years) and 53.5% were females.

Dyslipidemia screening was most frequently recorded (64.4%), followed by hypertension screening (50.8%). The least often recorded were tobacco (36.3%) and alcohol screening (40.5%) (Table 1). Significantly higher screening levels were recorded in patients studied in Catalonia than in Navarre, particularly in the case of alcohol (2.8 times higher) and tobacco use (more than double).

**Table 1:** **Individual, general practitioner (GP), and primary health care (PHC) team characteristics of the total visited study population and by region (Catalonia, Navarre), 2008**

	**Total sample**		**Catalonia**		**Navarre**	
	**N = 468940**		**n = 288479**		**n = 180461**	
**Individual**	**n**		**n**		**n**	
Age, years, mean (SD)		49.0 (19.3)		48.6 (19.3)		49.7 (19.2)
Age						
<45 years		220803 (47.1)		139193 (48.3)		81610 (45.2)
≥ 45 years and <65 years		138321 (29.5)		82720 (28.7)		55601 (30.8)
≥ 65 years		109816 (23.4)		66566 (23.1)		43250 (24.0)
Sex, female		251107 (53.5)		155775 (54.0)		95332 (52.8)
Number of visits, mean (SD); median (IQR)		8.5 (9.4); 6.0 (3–11)		8.7 (9.7); 6.0 (3–11)		8.0 (8.9); 5.0 (3–10)
Number of visits^a^						
[1,2]		111837 ( 23.8)		67143 ( 23.3)		44694 (24.8)
[3-5]		117009 (25.0)		70147 (24.3)		46862 (26.0)
[6-10]		114303 (24.4)		70283 (24.4)		44020 (24.4)
≥ 11		125791 (26.8)		80906 (28.0)		44885 (24.9)
Number of health problems, mean (SD); median (IQR)^a^		8.9 (7.0); 7.0 (4–12)		6.5(5.4); 5.0 (3–9)		12.8 (7.5); 11.0 (7–17)
Number of health problems						
[0–3]		107007 (22.8)		97124 (33.7)		9883 (5.5)
[4–6]		100888 (21.5)		74225 (25.7)		26663 (14.8)
[7–11]		128123 (27.3)		72957 (25.3)		55166 (30.6)
≥ 12		132922 (28.3)		44173 (15.3)		88749 (49.2)
Arterial hypertension		95737 (20.4)		59347 (20.6)		36390 (20.2)
Dyslipidaemia		85081 (18.1)		48369 (16.8)		36712 (20.3)
Obesity		68331 (14.6)		47192 (16.4)		21139 (11.7)
Tobacco use						
Nonsmoker		350862 (74.8)		202908 (70.3)		147954 (82.0)
Exsmoker		37457 (8.0)		27646 (9.6)		9811 (5.4)
Smoker		80621 (17.2)		57925 (20.1)		22696 (12.6)
At-risk drinker		10112 (2.2)		8149 (2.8)		1963 (1.1)
**General practitioner**	426		252		174	
Age (yrs), mean (SD)	423	46.4 (8.8)	249	46.1 (8.7)	174	46.9(9.1)
Sex, female	426	240 (56.3)	252	142 (56.3)	174	98 (56.3)
Number of patients assigned, mean (SD)	426	1452 (307)	252	1534 (286)	174	1334 (299)
Average number of patients’ health problems, mean (SD); median (IQR)	426	7.6 (3.5); 7.2 (4.4-10.8)	252	5.4 (2.4); 4.9 (3.5-6.7)	174	10.8 (2.1); 11 (9.3-12)
Coverage, mean (SD)^b^	426	76.0 (6.1)	252	74.9 (6.0)	174	77.5 (6.0)
Percentage of patients > = 65 years, mean (SD)	426	19.6 (7.2)	252	19.1 (7.0)	174	20.2 (7.5)
Teaching mentor (Yes)	424	91 (21.5)	252	61 (24.2)	172	30 (17.4)
Time with the same patient list (yrs), mean (SD); median (IQR)	419	10.1 (9.1); 7.0 (3–17)	246	10.7 (8.3); 8.0 (3–17)	173	9.3 (10.1); 5.0 (2–12)
**PHC team**	44		22		22	
Number of professionals, mean (SD); median (IQR)		10 (4.2); 9 (6–13)		11.4 (4.5); 11.5 (8–15)		7.9 (3); 7.5 (6–9)
Average number of patients, mean (SD)		1423 (193.3)		1514 (184.3)		1332 (158.4)
Average number of patient health problems, mean (SD); median (IQR)		8.1 (3.2); 8.8 (5–10.9)		5.5 (2.1); 5.1 (3.9-6.4)		10.7 (1.4); 10.8 (9.7-11.9)
Average coverage, mean (SD)^b^		77 (4.4)		75.8 (4.7)		78.2 (3.9)
Average percentage of patients > = 65, mean (SD)		19.5 (4.5)		18.9 (3.6)		20.2 (5.2)
Type						
Urban		22 (50.0)		13 (59.1)		9 (40.9)
Semiurban		10 (22.7)		7 (31.8)		3 (13.6)
Rural		12 (27.3)		2 (9.1)		10 (45.5)
Adherence to PAPPS (Yes)		11 (25.0)		6 (27.3)		5 (22.7)
Teaching centre (Yes)		12 (27.3)		7 (31.8)		5 (22.7)
Years of electronic health records data, mean (SD); median (IQR)		5.2 (1.6); 5.0 (4.0-6.8)		4.9 (1.8); 4.0 (4–6.25)		5.5 (1.3); 5.0 (4–7)
**Outcomes**						
Hypertension screening	373203	189746 (50.8)	229132	136853 (59.7)	144071	52893 (36.7)
Dyslipidaemia screening	383859	247380 (64.4)	240110	164481 (68.5)	143749	82899 (57.7)
Obesity screening	391075	162864 (41.6)	235595	114836 (48.7)	155480	48028 (30.9)
Tobacco use screening	350862	128561 (36.6)	202908	96715 (47.7)	147954	31846 (21.5)
Alcohol screening	458828	185851 (40.5)	280330	150969 (53.9)	178498	34882 (19.5)

Abbreviations: *IQR* interquartile range, *PAPPS* Program of Prevention and Health Promotion Activities, *SD* standard deviation.

Values are no. (%) unless otherwise indicated.

The variables for GP and PHC team are calculated on the basis of the assigned population. The PHC team averages are calculated as the mean of related GP variables.

^a^The number of visits and number of health problems are categorized by quartiles of the patient population.

^b^Coverage means percentage of assigned patients visited.

The median number of visits was 6 (interquartile range [IQR] = 3-11). The number of recorded health problems per patient was higher in patients studied in Navarre (median = 11, IQR = 7-17) than in Catalonia (median = 5, IQR = 3-9). On the other hand, a higher prevalence of obesity, smokers, exsmokers, and at-risk drinkers was observed in patients studied in Catalonia, and a smaller number of patients with dyslipidemia.

GPs’ mean age was 46.4 years and 56.3% were women. The average number of assigned individuals was greater for GPs in Catalonia, while the annual coverage per GP was slightly higher in Navarre. In Navarre, 45.5% of the PHC teams studied were rural, compared to 9.1% in Catalonia. A mean 5.2 years of electronic health records were available.

All individual characteristics appeared to significantly contribute to all the models, except for at-risk drinker, which was not associated with screening for hypertension or obesity, and sex, which was not associated with alcohol screening (Table 2). On average, independent of the type of screening, factors that were positively associated with higher probability of screening were being older, being a current or former smoker, making a higher number of PHC visits, or having a diagnosis of hypertension, dyslipidemia, or obesity. Except for tobacco use, women were more likely than men to be screened.

**Table 2:** **Fixed effects of covariates on the odds of being screened (Multilevel logistic regression model [N = 468940, no. PHC teams = 44, no. general practitioners = 426], Catalonia and Navarre, 2008)**

	**Hypertension screening (50.8%)**		**Dyslipidaemia screening (64.4%)**		**Obesity screening (41.6%)**		**Tobacco use screening (36.6%)**		**Alcohol screening (40.5%)**	
	**n = 373203**		**n = 383859**		**n = 391075**		**n = 350862**		**n = 458828**	
**Fixed parameters** ^**a**^	**OR (95% CI)**	**P-value**	**OR (95% CI)**	**P-value**	**OR (95% CI)**	**P-value**	**OR (95% CI)**	**P-value**	**OR (95% CI)**	**P-value**
**Individual**										
Age		<.0001		<.0001		<.0001		<.0001		<.0001
<45 (Ref.)	1		1		1		1		1	
≥ 45- < 65	0.90 (0.80-1.01)		1.88 (1.76-2.01)		1.32 (1.18-1.47)		1.51 (1.30-1.75)		1.79 (1.56-2.04)	
≥ 65	2.12 (1.88-2.38)		2.56 (2.39-2.75)		1.94 (1.74-2.17)		1.90 (1.63-2.21)		2.85 (2.49-3.26)	
Sex (male vs. female)	0.85 (0.83-0.87)	<.0001	0.58 (0.55-0.61)	<.0001	0.84 (0.82-0.86)	<.0001	1.11 (1.09-1.13)	<.0001		
Number of visits		<.0001		<.0001		<.0001		<.0001		<.0001
[1–2] (Ref.)	1		1		1		1		1	
[3–5]	1.72 (1.63-1.81)		1.80 (1.69-1.91)		1.63 (1.54-1.73)		2.16 (2.06-2.25)		2.10 (1.95-2.26)	
[6–10]	2.65 (2.51-2.80)		2.64 (2.48-2.81)		2.47 (2.33-2.61)		3.55 (3.40-3.72)		3.78 (3.51-4.07)	
≥ 11	4.55 (4.30-4.81)		3.66 (3.43-3.90)		3.63 (3.42-3.85)		5.73 (5.47-6.00)		6.62 (6.14-7.14)	
Number of health problems		<.0001		<.0001		<.0001		<.0001		<.0001
[0–3] (Ref.)	1		1		1		1		1	
[4–6]	1.30 (1.23-1.37)		1.52 (1.40-1.64)		1.29 (1.25-1.34)		1.11 (1.08-1.14)		1.10 (1.01-1.19)	
[7–11]	1.42 (1.35-1.50)		2.10 (1.94-2.27)		1.40 (1.36-1.45)		0.99 (0.97-1.02)		1.01 (0.93-1.10)	
≥ 12	1.63 (1.54-1.73)		3.12 (2.86-3.39)		1.51 (1.45-1.57)		0.88 (0.85-0.91)		0.91 (0.83-0.99)	
Arterial hypertension	-		2.55 (2.42-2.69)		3.13 (2.81-3.49)	<.0001	2.01 (1.89-2.13)	<.0001	2.21 (1.99-2.47)	<.0001
Dyslipidaemia	1.77 (1.70-1.83)	<.0001			1.60 (1.54-1.66)	<.0001	1.40 (1.34-1.45)	<.0001	1.40 (1.35-1.45)	<.0001
Obesity	2.45 (2.23-2.69)	<.0001	2.07 (1.94-2.22)		-	<.0001	1.60 (1.53-1.67)	<.0001	1.83 (1.71-1.93)	<.0001
Tobacco use		<.0001		<.0001		<.0001				<.0001
Nonsmoker (Ref.)	1		1		1		-		1	
Exsmoker	2.17 (2.01-2.35)		2.14 (1.98-2.30)		3.51 (3.05-4.05)		-		1.85 (1.71-2.00)	
Smoker	1.63 (1.52-1.75)		1.42 (1.33-1.51)		2.18 (1.90-2.50)		-		1.43 (1.33-1.54)	
At-risk drinker			1.35 (1.26-1.44)	<.0001			1.40 (1.26-1.55)	<.0001	-	
**General practitioner**										
Sex (male vs. female)			0.90 (0.83-0.98)	0.013			0.83 (0.72-0.96)	0.012		
Average number of patients’ health problems^b,c^	1.39 (1.18-1.65)	<.0001	1.29 (1.15-1.45)	<.0001	1.26 (1.03-1.54)	0.023	1.49 (1.17-1.90)	0.001		
Coverage^d,e^	0.92 (0.86-0.98)	0.010					0.87 (0.79-0.96)	0.005		
**PHC team**										
Region (Navarre vs Catalonia)	0.45 (0.36-0.55)	<.0001	0.44 (0.34-0.56)	<.0001	0.60 (0.43-0.82)	0.001	0.27 (0.18-0.40)	<.0001	0.24 (0.17-0.35)	<.0001
Type										0.033
Urban (Ref.)									1	
Semiurban									1.65 (1.11-2.46)	
Rural									0.95 (0.62-1.46)	
Average coverage^d,e,f^									0.75 (0.62-0.91)	0.004
Average percentage of patients ≥ 65^e,f^			0.84 (0.73-0.97)	0.015	0.79 (0.66-0.94)	0.010			0.75 (0.62-0.90)	0.002

Abbreviations: *CI* confidence interval, *GP* general practitioner, *OR* odds ratio, *PHC team* Primary health care team, *Ref.* Reference.

Dash indicates covariate not considered in that analysis. The models included the significant covariates (p < 0.05).

^a^For the other dichotomous covariates, the reference category was ‘no’.

^b^The continuous number of health problems covariate at GP-level was centred at its PHC team mean.

^c^OR for average number of health problems by GP based on a 4 percentage units change.

^d^Coverage means percentage of assigned patients visited.

^e^OR for coverage and average percentage of patients over 65 years based on 5 percentage units change.

^f^The continuous PHC team-level covariates were centred at their grand mean.

Patients with hypertension, smokers, and exsmokers were more likely to be screened for obesity; those with dyslipidemia or obesity were more often screened for hypertension. The odds of being screened for hypertension, dyslipidemia, and obesity increased significantly as the number of health problems increased; the highest odds were found for dyslipidemia screening. Being an at-risk drinker was positively associated only with the odds of being screened for dyslipidemia and for tobacco use.

On average, the patient attended by a female GP was more likely to be screened for dyslipidemia and tobacco use. Individuals assigned to GPs with higher coverage were 8% less likely to be screened for hypertension and 13% less likely for tobacco screening. Moreover, the covariate “average number of health problems by GP” had a positive contextual effect in all cases except for alcohol screening.

At the PHC team level, the contextual variable “region” was strongly associated with all screenings, greatly reducing variance at this level in all models. On average, individuals in Navarre were less likely to be screened than those in Catalonia, with the lowest probability in alcohol screening (78% lower), followed by tobacco screening (73% lower). Being assigned to a PHC team with a higher average percentage of assigned individuals older than 65 years was associated with lower odds of dyslipidemia, obesity, and alcohol screening.

Table 3 shows measures of variation in the odds of being screened. Empty models showed a significant random effect, indicating that much of the variation in screening between GPs and PHC teams was related to the respective contextual effects. The smallest variability was observed for dyslipidemia screening. Alcohol screening varied substantially by GP and PHC team. Hypertension screening had the greatest explained variability (PCV = 86.3%) at the team level, followed by alcohol screening (PCV = 81.0%).

**Table 3:** **Random effects of covariates from models on Table** 2 **at the GP and PHC team level on the odds of being screened**

	**Hypertension screening**	**Dyslipidaemia screening**	**Obesity screening**	**Tobacco use screening**	**Alcohol screening**
	**Variance (SE)**	**Variance (SE)**	**Variance (SE)**	**Variance (SE)**	**Variance (SE)**
**Between PHC team level**					
Intercept^a^	0.052 (0.030)	0.112 (0.037)	0.139 (0.059)	0.330 (0.092)	0.136 (0.061)
Age slope	0.063 (0.012)	0.019 (0.004)	0.055 (0.010)	0.110 (0.019)	0.085 (0.015)
Sex slope		0.011 (0.003)			
Number of visits slope	0.011 (0.002)	0.018 (0.003)	0.013 (0.002)		0.021 (0.004)
Number of health problems slope	0.011 (0.002)	0.028 (0.005)			0.026 (0.004)
Hypertension slope	-		0.052 (0.013)		0.050 (0.014)
Dyslipidaemia slope		-			
Obesity slope	0.035 (0.011)	0.014 (0.005)	-		0.011 (0.004)
Smoker slope	0.020 (0.005)	0.017 (0.004)	0.095 (0.017)	-	0.020 (0.005)
**Between GP level**					
Intercept^a^	0.125 (0.018)	0.088 (0.012)	0.371 (0.036)	0.196 (0.043)	0.400 (0.041)
Age slope	0.056 (0.004)	0.018 (0.003)	0.062 (0.005)	0.108 (0.008)	0.079 (0.006)
Sex slope	0.019 (0.002)		0.015 (0.002)		
Number of visits slope	0.019 (0.002)		0.021 (0.002)	0.064 (0.004)	0.036 (0.003)
Number of health problems slope		0.013 (0.002)	0.021 (0.002)		0.015 (0.002)
Hypertension slope	-	0.067 (0.008)	0.054 (0.007)	0.160 (0.014)	0.074 (0.008)
Dyslipidaemia slope	0.029 (0.004)	-	0.042 (0.005)	0.053 (0.006)	0.030 (0.004)
Obesity slope	0.048 (0.007)	0.014 (0.004)	-	0.063 (0.007)	0.038 (0.005)
Smoker slope	0.027 (0.003)		0.047 (0.005)	-	0.058 (0.005)
Drinker slope				0.093 (0.028)	-
**Variances of the empty model** ^**b**^					
Between PHC teams	0.380 (0.088)	0.165 (0.039)	0.319 (0.078)	0.659 (0.151)	0.918 (0.206)
Between GPs	0.229 (0.017)	0.129 (0.010)	0.359 (0.026)	0.357 (0.026)	0.384 (0.028)
**PCV** ^**a,c**^					
Between PHC teams	86.3%	32.2%	56.4%	49.9%	85.2%
Between GPs	45.4%	31.8%	−3.3%	45.1%	−4.2%

Variances estimated in logits.

Abbreviations: *GP* general practitioner, *PCV* proportional change in variance, *PHC team* Primary health care team, *SE* standard error.

Dash indicates covariate not considered in that analysis.

The models included the variance of the random intercepts and slopes that significantly varied between GP and PHC team results.

^a^As the variance is a function of individual characteristics that vary randomly, the values in the table are for the intercepts (for individuals with characteristics at their reference values).

^b^Empty model: model with no covariates at either level, i.e. only the intercepts and the random parameters.

^c^PCV: The proportional change in variance expresses the change in the PHC team or GP level variance between the empty model and the final model.

The estimated variances of the individual characteristics suggested variability between GPs and between PHC teams in the effect of these covariates on the recorded screening. The magnitude of the association between screening and the individual characteristics that did not vary randomly at the GP or PHC team level was similar for all GPs or PHC teams.

## Discussion

The recorded screening ranges from 36.6% for tobacco to 64.4% for dyslipidaemia, with major differences between the 2 Spanish regions studied. These results are similar to other studies based on electronic databases [21,35]. Nonetheless, they are lower than results based on self-reports by health professionals [14,36] and patients [37]. Some of the problems of electronic databases are well known: underreporting during the first years of implementation, variability resulting from heterogeneity in coding, using open-text fields to record activity without linking it to a diagnosis, etc. [18,35,38,39]. All of these may explain disparities between studies. Despite the progressive increase in the recording of prevention activities [10], PHC screening activity remains low and can be greatly improved, especially with respect to tobacco and alcohol use. Advice on drinking behaviour is least often provided, probably due to a reluctance to ask patients about it unless there are clear signs of risky drinking behavior [40].

Our results agree in part with other studies in which practitioners from large urban areas reported more prevention services involving alcohol and drugs, while respondents in rural areas reported fewer screening procedures [15]. The studied PHC teams in Catalonia were more urban and their patients had a higher prevalence of all screenings, but especially for tobacco and alcohol use, than those in Navarre.

The most prevalent screening is for hypertension and dyslipidemia, which have the lowest unexplained variability between PHC teams and GPs, respectively, after adjusting for individual and contextual factors. A possible explanation is that these screenings, primarily related to the prescription of medications, are easier and preferred over lifestyle modification activities by some GPs [41]. On the other hand, tobacco and alcohol screening had the highest variability between PHC teams and GPs, respectively, that could not be explained by the contextual factors studied.

Overall, the odds of being screened were higher for women, older patients, those with more comorbidities, more cardiovascular risk factors, and more frequent office visits, and those assigned to a female GP, a GP with a lower patient load, or a PHC team with a lower percentage of patients older than 65 years. Region was the most important contextual factor at the PHC team level.

Morbidity was positively related to screening for hypertension, dyslipidemia, and obesity, as in other studies [12,17], showing that GPs take a more proactive approach to screening in patients with more pathologies. Regardless of the type of screening, patients with previously identified cardiovascular risk were more likely to be screened, perhaps due to the need to obtain information to calculate cardiovascular risk and determine appropriate treatment. In the case of at-risk drinkers, the only associations observed were with screening for dyslipidemia and for tobacco use, reflecting the approach to preventing consumption of addictive substances.

At the GP level, female GPs were more likely to screen for dyslipidemia levels and tobacco use, as in other studies of prevention activities [14-16,42,43]. Our study showed that increased patient coverage is associated with less screening, specifically hypertension and tobacco, as in other studies [12]. Similarly, at the PHC team level, having a high percentage of elderly patients was negatively associated with some screening activities [12]. This may be due to the increased work load and lack of time for carrying out preventive services that is perceived by PHC professionals [44].

At the PHC team level, contextual variables better explained major variability (more than 80% in the case of hypertension and alcohol), compared to the GP-level variables. The larger contextual PHC team-level effect was determined by the region. Possible differentiating factors include the software used by each region, because software design can determine what health professionals record [18,45], and organizational aspects inherent to the different health care policies in each region, such as economic incentives to conduct certain prevention activities, the rurality of the region, or sociocultural and socioeconomic aspects that affect individual behaviors. With regard to financial incentives, evidence suggests that they might be effective in changing the practice of healthcare professionals [46]. However, a lower level of screening was recorded in PHC teams from Navarre, where they had more incentives related to the studied screenings. This discrepancy may be explained by the variable “region”, which could act as a proxy for other important unobserved organizational and socioeconomic variables.

### Limitations and strengths

Our study has several limitations that must be acknowledged. It was based on a registry of daily clinical activity at the point when computerization of PHC health records had just begun to mature. The acquisition of good recording habits and the changes that occurred in the software over time could have affected the recording of clinical activity [39,47]. Finally, available programs did not allow adequate recording of the activities conducted by nursing professionals, despite their important role in prevention [10].

Due to differences in the implementation of electronic health records and the availability of data only 2 regions of Spain were included in the study. Future studies, with more regions, are needed to estimate the association between region-specific characteristics and screening. Other factors should be factored in to improve the quality of data collection: 1) Training of basic computer skills to health professionals; 2) Training of health professionals to adequately use and to keep up to date with the ECR; 3) Incentives, financial and otherwise, to increase the motivation of health professionals toward achieving a better completeness and quality of data. In addition, harmonization of variables and codification systems should be improved to enable information-system interoperability and data sharing for research [48].

Major strengths of the present study include its large sample size and multilevel random slopes. The large sample size drawn from REGIPREV, a database specífically focused on prevention activities, provided a broad view of PHC screening implementation. A multilevel approach allows us to separate the potential sources of variability (individual, GP and PHC team) and to control for clustering effects. The random slopes analysis contributes to examining whether the PHC team or GP environment as a whole would modify individual-level associations, without specifying any contextual factors. Moreover, it may show whether contextual influences have a different impact on screening for certain groups of individuals [32].

Variation remained statistically significant at the PHC team and GP level, even after accounting for individual and contextual factors. Future research should explore whether other individual factors (e.g., variables specific to each screening) and contextual features (such as factors linked to PHCT organization, changes in the software, nurses assigned to the patient, reminder alerts or feedback to GPs concerning prevention activities, etc.) may account for variation in the screening registry. Moreover, the random slopes analysis would allow the examination of contextual effects that pertain to specific groups of people and of cross-level interactions to establish PHC team-individual or GP-individual causal pathways.

## Conclusions

Low levels of implementation of the studied screening activities were observed in PHC, especially with respect to tobacco and alcohol use. At the individual level, more active strategies are needed for young people and for opportunistic screening (i.e., taking advantage of visits for other reasons) of those with few health problems who seldom see a doctor. With respect to health professionals, health policies are needed that limit the doctor:patient ratio permitting more time for preventive services. Screening related to lifestyles showed higher variability. Strategies such as training PHC professionals in approaches to lifestyle changes, provision of incentives and better screening protocols are needed [41].

In addition, information systems must continue to mature [48], improving data recording by PHC professionals and homogenizing the differences that exist between systems, especially when these have repercussions for the quality of the information provided. There is a need for further research that includes more regions and assesses additional individual and contextual factors.

## Additional file

Additional file 1: Table S1. List of primary health care teams that volunteered to participate in the study, by region (Catalonia, Navarre).
