# Supplementary material for: Variability in screening prevention activities in primary care in Spain: a multilevel analysis
Source: BMC Public Health. 2015 May 7;15:473. doi: 10.1186/s12889-015-1767-5 (PMC4440275; doi:10.1186/s12889-015-1767-5)
Supplement: Supplementary file 1 [file 12889_2015_1767_MOESM1_ESM.doc]

Supplementary table: List of Primary Health Care Teams that volunteered to participate in the study, by region (Catalonia, Navarre)

Catalonia	Navarre
Balàfia-Pardinyes-Secà de Sant Pere (Lleida 4)	Il Ensanche (Pamplona)
Tarragona 5 (St Pere i St Pau)	Ansoain
Deltebre	Azpilagaña (Pamplona)
Salt	Berriozar
Olot	Buñuel
Roses	Chantrea (Pamplona)
Gornal (L'Hospitalet de Llobregat 11)	Cintruénigo
Florida Sud (L'Hospitalet de Llobregat 8)	Cizur
Penedès Rural	Corella
Piera	Ermitagaña (Pamplona)
El Prat de Llobregat 3	Estella
Sant Andreu de la Barca	Etxarri-Aranatz
Lluís Millet (Esplugues de Llobregat 2)	Huarte
Sant Sadurní d'Anoia	Leitza
La Mina (St Adrià de Besòs 2)	Lesaka
Badia del Vallès	Lodosa
La Serra (Sabadell 7)	Milagrosa (Pamplona)
Sant Vicenç de Castellet	Peralta
Via Roma (Barcelona 2-B)	Rochapea (Pamplona)
Chafarinas (Barcelona 8-I)	San Adrián
Barcelona Horta (Barcelona 7-D)	Santesteban
Vilassar de Dalt	Villava
